# GatorByte – An Internet of Things-Based Low-Cost, Compact, and Real-Time Water Resource Monitoring Buoy

**DOI:** 10.1016/j.ohx.2023.e00427

**Published:** 2023-05-10

**Authors:** Piyush Agade, Eban Bean

**Affiliations:** Agricultural and Biological Engineering Department, University of Florida, 1741 Museum Rd, Gainesville, FL 32611, USA

**Keywords:** Environmental Internet of Things, Physiochemical water quality monitoring, Urban water quality monitoring, Spatiotemporal water quality monitoring, Low-cost sensors

## Abstract

Conventional water resource monitoring systems are usually expensive, have a low-temporal resolution, and lack spatial dimension entirely. These systems are typically available as stations or handheld devices. Pinpointing sources of pollution using these systems can be difficult. This project involves developing a high-resolution free-flowing monitoring buoy that records spatiotemporal water-quality data in flowing stream environments. The system is highly customizable, and even users with limited experience in programming or electronics can tailor GatorByte to their needs. The platform includes a data logger, a cloud-based server, and visualization tools. The data logger uses low-cost sensors, electronic peripherals, a 3D-printed enclosure, and printed circuit boards, with a total cost per unit under $1,000 USD. The data logger uses an NB-IoT-capable Arduino for real-time reporting and visualizing sensor data. The GatorByte records physiochemical water metrics – pH, temperature, dissolved oxygen, electroconductivity, and the current location of the buoy using a GPS module. The data logger also includes micro-SD storage and a Bluetooth module for on-field diagnostics. Using the GatorByte buoy, the collection of variations in water quality data in temporal as well as spatial dimensions can be achieved cost-effectively and reliably, enabling quick detection and resolution of pollution events.

## Graphical abstract

**Figure:**
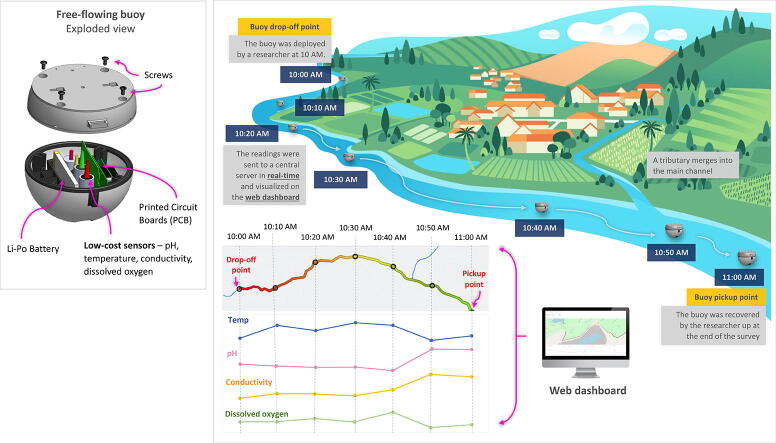

## Specifications table

**Table:** 

Hardware name	GatorByte buoy
Subject area	Environmental, Planetary and Agricultural Sciences
Hardware type	Field measurements and sensors
Closest commercial analog	No commercial analog is available
Open-Source License	CERN Open Hardware License v2.0 (PCB and Hardware design), GNU GPL v3.0 (Firmware), GNU AGPL v3.0 (MQTT Broker, Server, and Dashboard)
Cost of Hardware	$950 USD (buoy), $2 USD per month (cellular service)
Source File Repository	https://doi.org/10.17632/csf6hhvy3b.1 (Web application and dashboard), https://doi.org/10.17632/y2gc2fwj5b.1 (Firmware files), https://doi.org/10.17632/gjbkxb74dw.1 (Enclosure and PCB CAD files)

## Hardware in context

The GatorByte platform constitutes a suite of hardware and software modules aiming to provide water resource managers (WRMs) with an inexpensive, real-time water quality monitoring solution. The modular hardware and software design [1] allows the user to quickly deploy the buoy with supported sensors and peripherals. The electronics hardware consists of a microcontroller hat and a sensor baseboard. The modular design pattern allows users to add other environmental monitoring sensors and adapt the system to their requirements.

The platform aims to be open source, serviceable, reproducible, and easily customizable.

GatorByte’s sensing and data reporting is based on the prototype described in the widely reviewed Rao et al. [2]. The GatorByte uses low-cost, commercially available water quality sensors, which allow for real-time high-frequency measurements with acceptable accuracy (<5%, as per manufacturer’s specifications).

### Motivation

Many urban freshwater bodies have become impaired due to point and non-point sources of pollution. Pollutants such as sediments, pathogens, and nutrients are contributed by a variety of sources, including stormwater runoff, leaking wastewater infrastructure, and channel erosion have wreaked havoc on urban water bodies. These pollutants disturb the balance of nutrients, oxygen, and organic compounds, harming biota, and their habitat. Point sources of pollution can be mistakenly lumped under non-point source pollution and assumed to come from large areas due to a lack of high-frequency water quality data to locate change points. This issue is amplified during storm events when pollutants from urban areas are transported to downstream waters in a short period. This results in abrupt degradation of water quality which may not be picked up by low-frequency sampling or monitoring. Moreover, monitoring stations capable of high-frequency monitoring, if installed in downstream waters, will pick up short-lived water quality variations. However, the mixing of pollutants from various contributing areas will confound identification of the pollutant origin(s). Knowing the source location, or at least the entry point, of the pollutants is as important as quantifying the pollutants for mitigating the effects of pollution in a timely manner [3], [4]. Being able to locate the source of pollutants allows for direct mitigation at the source or point of entry. However, this can be challenging using current monitoring techniques, especially within complex urban watersheds.

Besides declining water quality across water resources, the state of Florida is also experiencing freshwater shortages [5], making the monitoring and conservation of water quality even more important. Algal blooms and red tide [6] are widely known water quality issues in the state of Florida due to excess nutrients from agriculture, urban stormwater, municipal and septic wastewater systems, and natural sources.

Conventional approaches to watershed assessment rely on monitoring and/or sampling at fixed points. This data is then used with hydrologic and water quality modeling to infer upstream pollutant sources. These monitoring systems do not record spatial water quality variations on their own. Personnel must manually translocate the measurements and independently record the location, which still requires each location to be accessible by personnel. This may introduce additional labor costs and efforts, inconvenience, risks to personnel, and measurement errors. Moreover, water quality can drastically change over short distances depending on relative flows and pollutant quantities, which cannot be detected using this approach. The GatorByte buoy was developed as a tool to specifically address this challenge and more directly locate changes in water quality. The buoy is designed to float along the water surface with the flow or currents of a water course while collecting geotagged water quality measurements. This enables the collection of high-density water quality measurements without the need for personnel to access each sampling point. This will enable water resource managers to target points or areas where pollutants are introduced into urban water systems more directly.

**Fig. 1:**
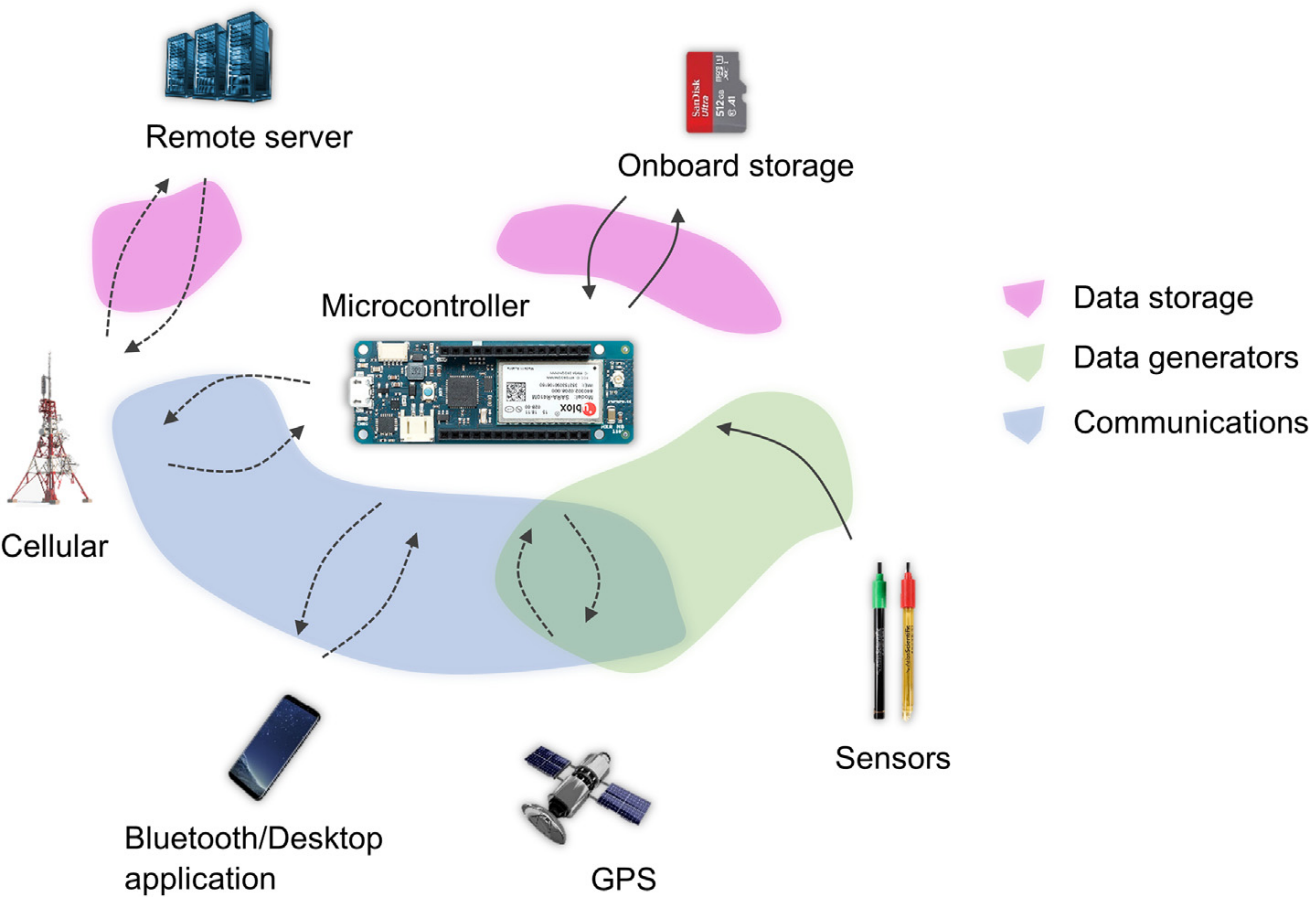
**Platform Overview –** Overview of the GatorByte platform and its components that includes the sensors, actuators, data locations, and communication systems.

The GatorByte platform (Fig. 1) uses off-the-shelf components, low-cost sensors, and a GPS module to geotag water quality data. The primary application of the platform is to perform high-frequency (up to 60 readings per hour) short-term (few hours to days) surveys in urban, or otherwise difficult-to-access, water bodies. The buoyant and self-orienting housing was specifically designed to facilitate migration through shallow channels while providing a recessed housing for the sensors to be protected from impacts and other hazards. Moreover, the compact form factor of the buoy housing enables monitoring of hard-to-access locations. This differentiates it from other similar water monitoring buoys (e.g., Trevathan and Schmidtke, and Trevathan and Nguyen [14], [15]) that are not designed to traverse and endure these conditions. The onboard cellular (LTE-M/NB-IoT) networking capabilities enable real-time reporting of sensor and GPS data that enable the users to deploy, track, and recover the unit.

Currently available solutions for field water quality measurements include deployable sondes (e.g., Hydrolab HL7, YSI EXO platforms, Eureka water probe’s EasyProbe) and hand-held units (e.g. YSI® Pro Quatro). These instruments require skilled personnel to operate and collect water quality data. Moreover, these devices use expensive sensors and electronic components which drive their costs into the thousands or tens of thousands of dollars. This is cost prohibitive to many water resource managers and lacks the integrated design to collect spatial water quality. In contrast, the GatorByte leverages low-cost sensors, and electronic components and adds a spatial dimension to the water quality data, resilient housing design, and a wide range of sensor support within an end-to-end water resource monitoring solution.

To summarize, there are currently limited options for low-cost water resource monitoring. Moreover, these solutions do not provide the ability to capture spatial changes in water quality. GatorByte aims to be a viable water resource monitoring solution to directly address this need.

### Related work

Several systems have been developed that address some of the issues with the commercially available water quality monitoring systems. RiverCore [7] and CavePearl [8] are two examples of recent projects that have done so and have similarities to GatorByte. However, RiverCore monitors water levels as part of a real-time flood warning system, while CavePearl is a water quality datalogger, being used in submerged cave systems. CavePearl does not report data in real-time and requires manual extraction of data.

O’Flynn et al. [9] presented a Zigbee-based wireless monitoring system (named SmartCoast) for monitoring the physiochemical characteristics of water resources. SmartCoast consists of wireless sensor nodes that record water quality metrics, including temperature, pH, conductivity, depth, phosphate, dissolved oxygen, and turbidity.

Nguyen et al. [10] proposed a cellular-based (GSM/GPRS) wireless sensor network to monitor pH and temperature in ponds. The authors also present strategies to minimize high-energy consumption and network losses of a remote monitoring system. The system is made of sensor nodes and central coordinator devices. The nodes read sensor data and relay the data to the coordinator node using Zigbee every 30 min. The coordinator node uploads the collected data from sensor nodes to a remote server using GPRS (General Packet Radio Service).

Yue et al. [11] proposed a solar-powered distributed Wireless Sensor Network (WSN) for monitoring oxygen density, pH, and turbidity. The sensor network architecture consisted of sensor nodes and a base station. The nodes and base station use Low-rate Wireless Personal Area Network (LR-WPAN) to communicate.

Lambrou et al. [12] developed a real-time in-line water quality monitoring system for water distribution systems based on Zigbee communication. They used pH, Oxidation Reduction Potential (ORP), electroconductivity, and turbidity sensors as primary parameters.

Udell et al. [13] developed a fast environmental sensing prototyping framework (Loom) to allow users to quickly create monitoring systems from a wide selection of supported sensors. The researchers have also designed a Printed Circuit Board (PCB), named Hypnos, that abstracts many critical and functional components, like Real Time Clock (RTC), SD logging module, sensor multiplexing circuitry, and coin cell battery on a condensed circuit board that users can use in their projects right off the box.

Trevathan et al. [14], [15], [16] developed a long-term remote water quality monitoring buoy. The buoy monitors lux, temperature, turbidity, pH, dissolved oxygen, and salinity. The buoy uses TinySine 3G cellular module to add cellular networking capabilities for reporting data, and the data is visualized using the ThingsBoard IoT dashboard. The buoy uses duty-cycling procedures and employs a solar panel to extend the operational life of the buoy.

Wickert et al. [17] proposed an Arduino-compatible family of data loggers (ALog) and introduced a modular firmware design allowing the expansion of sensor support. The system aimed to be low-cost, low-power, and lightweight.

Fuentes-Pérez et al. [18] developed a drifting buoy built using off-the-shelf components. The drifter uses a 9-axis accelerometer and Neo-8MT GPS module to estimate river flow velocity cost-effectively, in contrast to commercially available flow meters. The researchers provided an open software framework (SPart) to enable real-time visualization of the data. The drifter offers two modes of communication – GSM and Long-range Radio (LoRa). The researchers have used the MQTT protocol to upload data to the cloud.

Makhtar et al. [19] proposed a real-time water quality monitoring bench-top system based on Arduino Uno. The system monitors pH, turbidity, total dissolved solids (TDS), and temperature. The system shows the latest readings on an LCD screen. Users can connect to the device using a smartphone using Bluetooth and fetch the latest readings.

GatorByte leverages the technologies and methods adopted by the aforementioned researchers [7], [19], including sensor selection, characterization, communication technologies, power-saving strategies, and data integrity. The GatorByte platform builds upon their work by incorporating the tried and tested electronic components and water quality sensors, best data management practices, and power-saving techniques to furnish an inexpensive, customizable, open-source, compact, and resilient buoy capable of real-time reporting and visualization of spatiotemporal water quality data.

In summary, GatorByte’s objectives are:

1. Provide WRMs with a low-cost end-to-end water quality monitoring, and visualization solution.
2. Allow WRMs to understand spatiotemporal water quality variations in difficult-to-access streams, drainage systems, lakes, ponds, creeks, or rivers and allow a quicker and more direct determination of the location of the pollutant sources.
3. Make the GatorByte software and hardware open-source and modular for further expansion of the list of supported components and sensors.

## Hardware description

The GatorByte’s hardware has three major components as shown below.

a. Buoy enclosure
b. Printed Circuit Boards (PCB)
c. Data generators
d. Electronic components

### Buoy enclosure

**Fig. 2:**
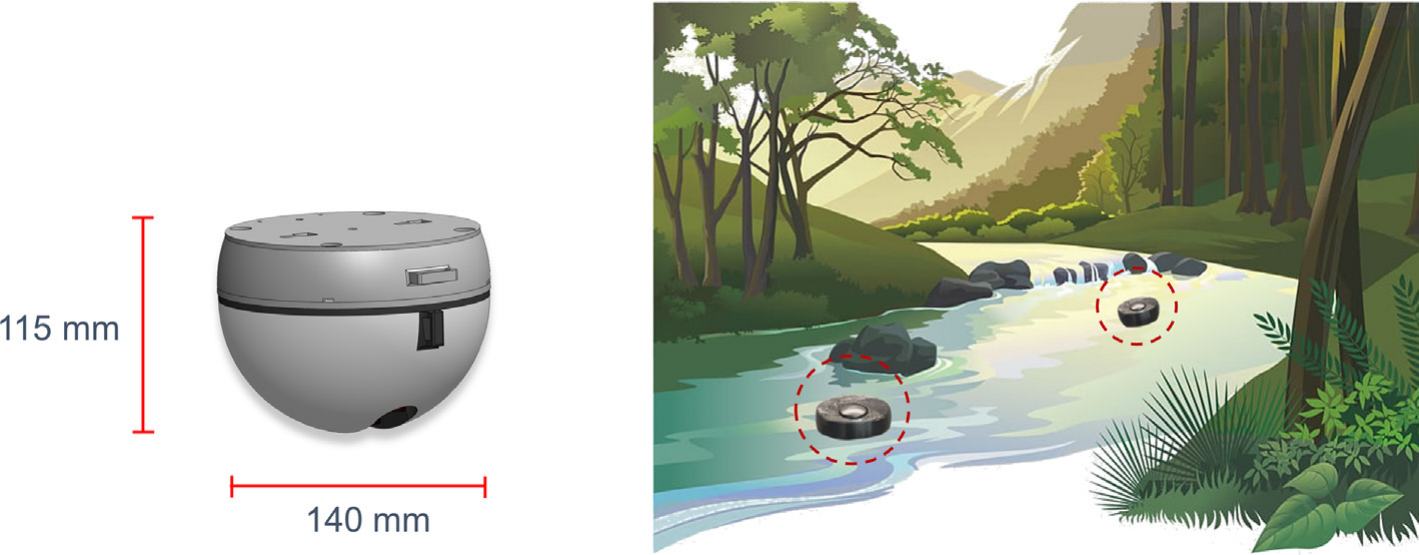
**Buoy dimensions and survey illustration –** The image on the left shows a GatorByte buoy’s 3D printed housing and its dimensions. The small form factor allows deployments in narrow or shallow waterways. The illustration on the right shows two GatorByte buoys surveying water quality in a stream.

The housing of the buoy is a 3D printable enclosure, 140 mm in diameter and 115 mm tall (Fig. 2). The enclosure was printed using an inexpensive Prusa i3 MK3s 3D printer for ∼$17 per enclosure, including sealing and other hardware. The enclosure has two parts: the bottom and the lid. The bottom enclosure houses the sensors, most of the electronic components, and the battery. While the antenna, indicator LED, and magnetic power switches are mounted on the lid.

**Fig. 3:**
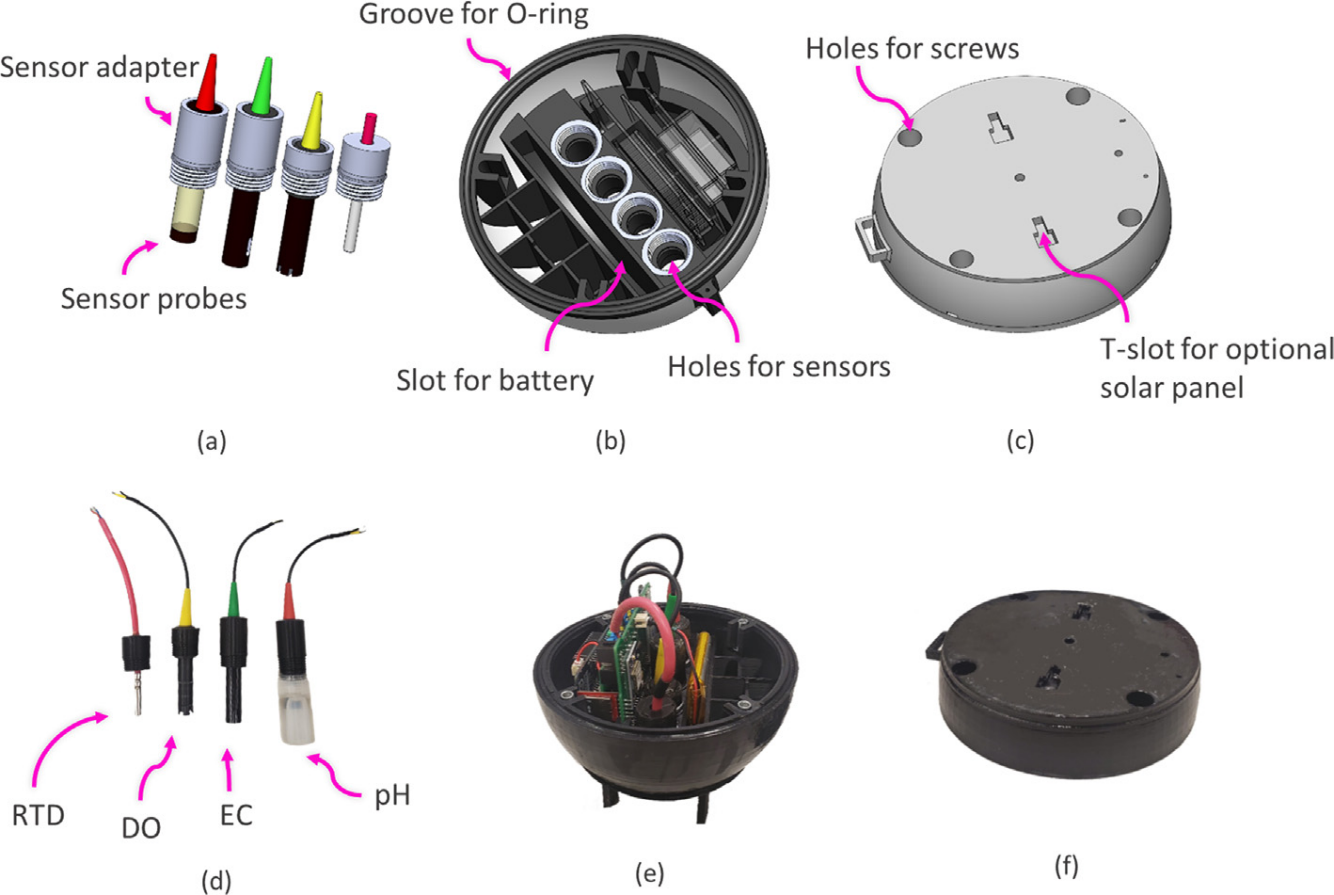
**Hardware CAD illustrations – a)** 3D models of the pH, EC, DO, and RTD sensors in their sensor adapters, **b)** the bottom enclosure showing the holes where sensor adapter thread in, slots for the PCB and the battery, **c)** the enclosure lid with holes for screws and T-slot for an optional solar panel attachment, **d)** the sensor probes attached to the adapters, **e)** an assembled buoy bottom enclosure with all the electronics, sensors, and battery. The enclosure is sitting on a 3D printed stand. **f)** The lid enclosure that attaches on top of the bottom enclosure with four metal screws.

The sensor probes, using a 3D printed adapter, thread into the bottom enclosure (Fig. 3a and b). The bottom housing is designed so that the tip of each sensor is recessed within the outer buoy radius (see recess on the bottom of the buoy in Fig. 2) to protect them from direct impact with channel stones, boulders, or otherwise. The sensor interface is sealed using O-rings to prevent leaks. Small weights are used as ballasts in the bottom enclosure to optimize vertical orientation and righting of the buoy in turbulent conditions. The interface between the enclosure lid and bottom is sealed using four press-fit metal screws and an O-ring (Fig. 3b and c). A spray sealant is typically applied before deployment as an extra step to avoid any water entering through the joint.

Three common and inexpensive filament materials and waterproofing treatments were evaluated during the development of the housing: PLA (Poly-lactic Acid), PET-G (Poly-ethylene Terephthalate, Glycol-treated), and ASA (Acrylonitrile Styrene Acrylate). These materials can be printed with commonly available, hobby-grade fused deposition modeling (FDM) 3D printers.

PLA deteriorates when exposed to moisture and sunlight. Hence, PLA is not recommended to fabricate parts that will be in contact with water and exposed to sunlight. PET-G is more resistant to water and sunlight compared to PLA and only requires moderate adjustments to printer settings. ASA filament is best suited for environmental IoT applications where water and sunlight are abundantly present. ASA has high resilience against UV, water, and other environmental factors. Hence, the GatorByte buoy uses ASA to fabricate its enclosure. Moreover, ASA prints can be sealed by treating the prints using Acetone vapors in a closed volume with air circulation [20] for better waterproofing. A polypropylene storage box was fitted with a moderate RPM fan to treat the GatorByte enclosure for this purpose. Application of three to five coats of spar urethane to ASA prints after acetone treatment for added resilience is recommended. Allow each coat to cure for 90 min before reapplication (or follow product recommendations).

### Printed circuit boards

**Fig. 4:**
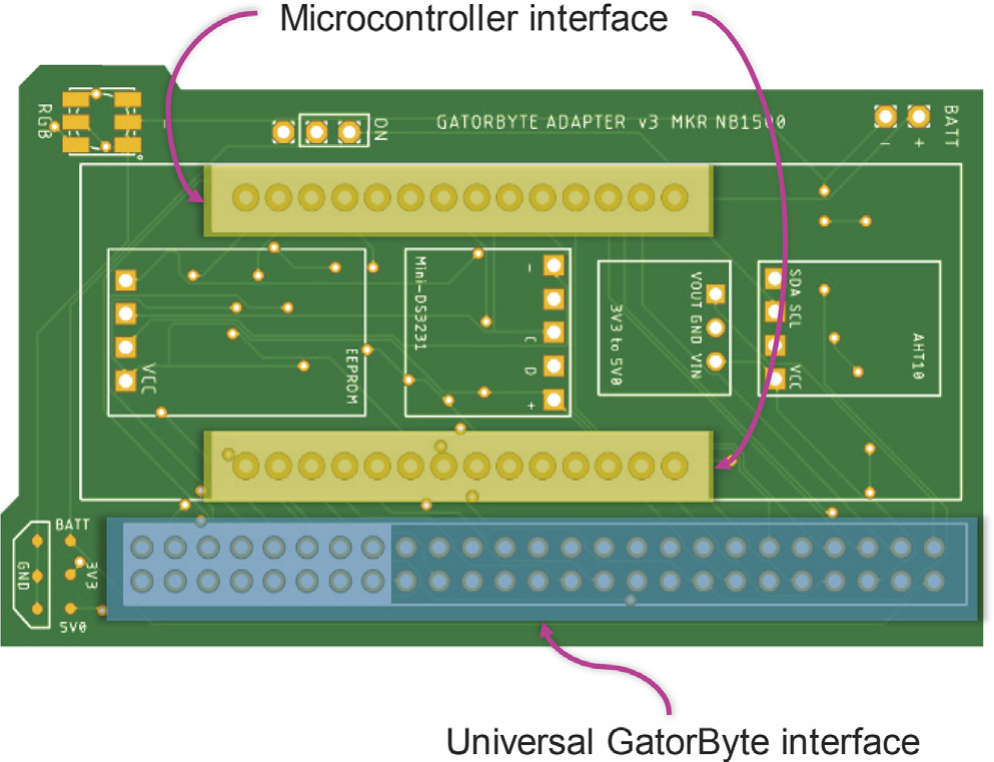
**Microcontroller hat CAD illustration –** The microcontroller hat schematic houses various electronic components, the microcontroller, and the universal GatorByte interface to connect to the sensor baseboard PCB.

**Fig. 5:**
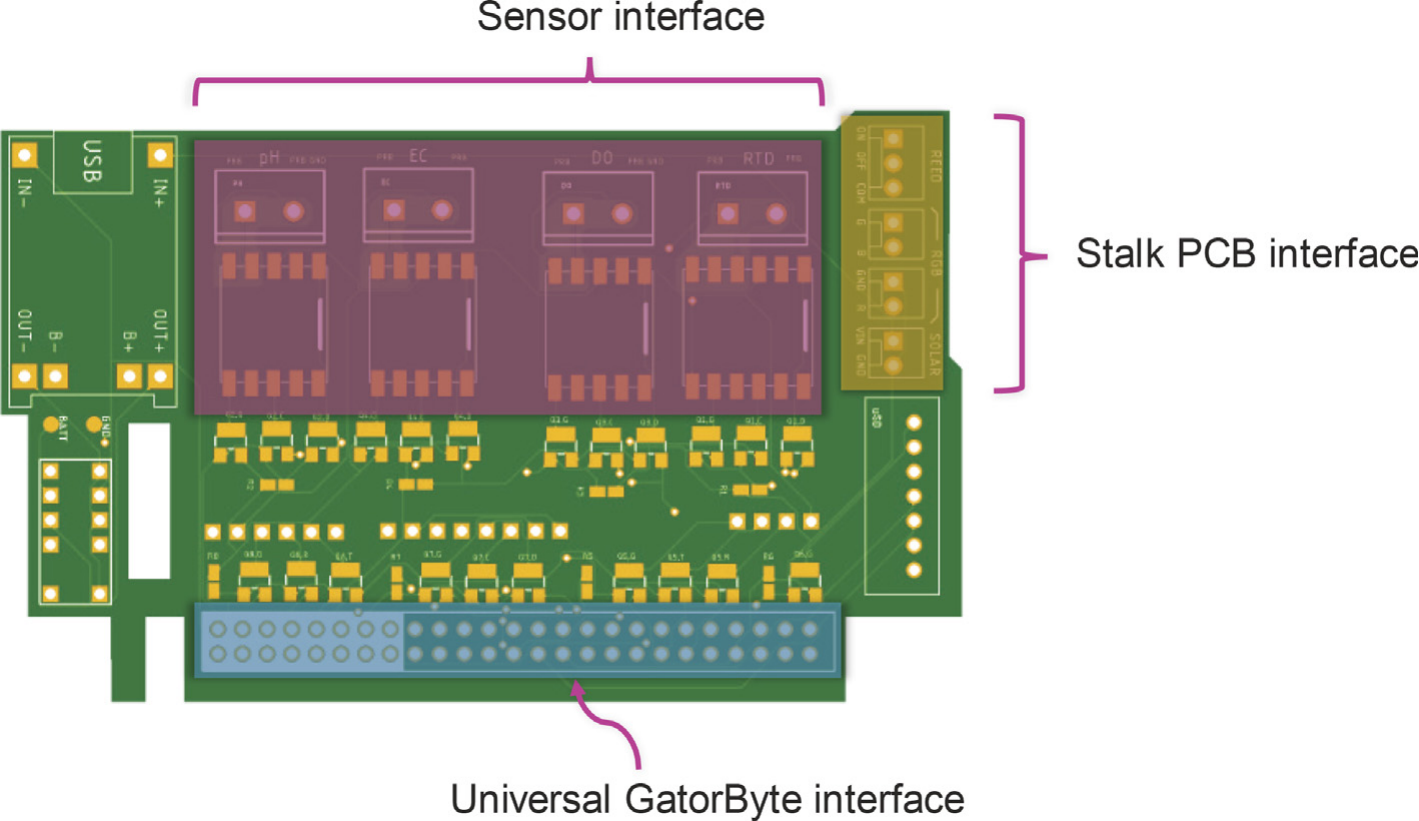
**Sensor baseboard CAD illustration –** The sensor baseboard schematic shows footprints for sensor interface ICs and connection terminals, the stalk PCB interface, and the universal GatorByte interface where the microcontroller hat plugs in.

**Fig. 6:**
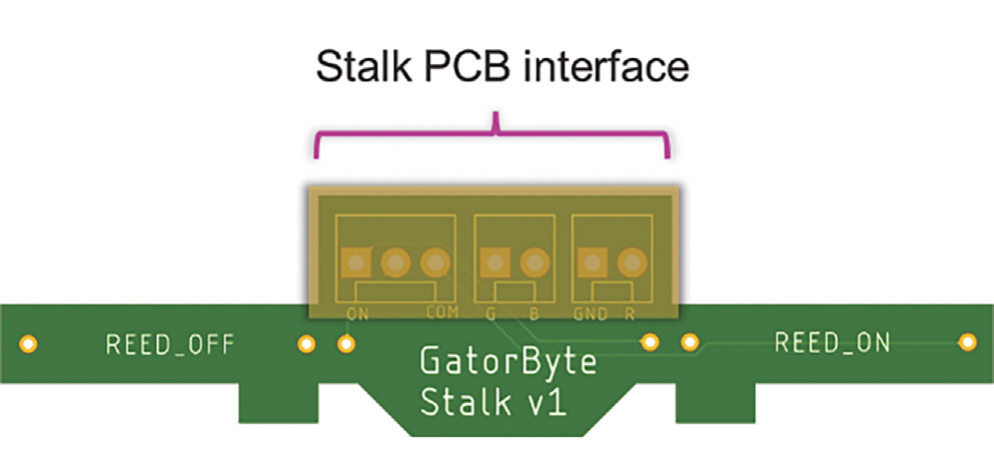
**Stalk PCB CAD illustration –** The stalk PCB houses the reed switches and the RGB indicator LED. This PCB connects to the sensor baseboard via the stalk PCB interface.

The GatorByte buoy has three modular circuit boards that interconnect with each other using the universal GatorByte interface (Fig. 4, Fig. 5, Fig. 6).

#### Microcontroller hat

The microcontroller hat houses the microcontroller board and the non-optional electronic components that are essential for the datalogger functions: Real-time clock (RTC), Electrically Erasable Programmable Read-only Memory (EEPROM), Internal temperature and humidity sensor module, I/O expander, and a 3.3 V to 5 V booster.

The I/O expander (SN74HC595 Shift register IC) can be configured as a 1:8 expander (1 pin to 8 pins expansion). GatorByte uses two shift-register ICs chained together to create a 1:16 pins I/O expander.

The voltage booster converts the 3.3 V from the microcontroller to 5 V. This device, while being inexpensive, expands the compatibility of the GatorByte to sensors and electronic peripherals that require 5 V to function (for example, DS1307 RTC). The user must include a bidirectional logic level shifter if such peripherals only support 5 V logic level.

#### Sensor baseboard

The GatorByte buoy’s sensor baseboard holds the Atlas Scientific sensor interface ICs and screw terminals for connecting temperature, pH, dissolved oxygen, and electroconductivity sensor probes. The circuit also houses an SD card module for local data storage, a Lithium-Polymer (Li-Po) battery charging module, a Bluetooth module, and a GPS module. The sensor baseboard uses the universal GatorByte interface where the microcontroller hat can be attached.

The sensor baseboard is a customizable circuit board that consists of electronic components specific to the objectives or requirements of the user. In other words, users can redesign a baseboard PCB and only include the sensors and peripherals they intend to use. For instance, if the objective is to track floating vegetation on a lake, users can solely include a GPS module besides mandatory peripherals like the battery charging module and micro-SD module.

#### Stalk

The stalk circuit consists of two magnetic (reed) switches that allow turning on and off the datalogger using a magnet, which minimizes the number of times the enclosure’s water-proof seal needs to be opened. This PCB also includes a multicolor Light-Emitting Diode (RGB LED) for indicating buoy status and diagnostics.

### Data generators (Sensor probes, RTC, and GPS modules)

The buoy uses low-cost, compact, lab-grade environmental sensors made by Atlas Scientific (Table 1). These sensors have a small form factor and are inexpensive, in turn allowing the buoy to be compact and keep the overall cost low. The sensors have a life of 1 to 2 years and require periodic calibration and proper storage for achieving the desired accuracy. The sensors were designed to be used in a laboratory setting. However, the GatorByte’s enclosure incorporates these sensors allowing them to be used in the field. The sensor probes attach to the enclosure with a 3D printed adapter (Fig. 3a). The probes are exposed to water through holes on the bottom of the enclosure but are protected by the buoy design which recesses the sensor tips above the bottom of the buoy. The enclosure also uses O-rings, Teflon tape, epoxy glue, and plumber’s grease to prevent water ingress at the interface.

**Table 1:** Sensors currently used in the GatorByte buoy.

Measurement	Units	Sensor	Interface IC	Product ID
Temperature	°C	Atlas Scientific PT-1000	OEM 2- Wire	PT-1000
pH	–	Atlas Scientific mini lab pH	OEM	ENV-20-PH
Electroconductivity	µS/cm	Atlas Scientific mini lab EC K 1.0	OEM	ENV-20-EC-K1.0
Dissolved oxygen	mg/L	Atlas Scientific mini lab dissolved oxygen	OEM	ENV-20-DOX
Relative Humidity and Temperature	% R.H., °C	Generic AHT-10 module	N/A	AHT-10

The buoy includes an AHT-10 temperature and relative humidity sensor to monitor the internal conditions of the enclosure. This data can indicate whether a slow leak is present, or if temperatures are outside the manufacturer’s recommended operating ranges. Additionally, GatorByte supports other sensors that communicate using I^2^C, UART, SPI protocols, or analog signals.

The u-blox Neo-6 M GPS module in the buoy provides location data (∼2m accuracy). The module requires 45 s to 5 min to acquire a location fix the first time it is powered on. The module has a coin-cell battery that allows it to store satellite information for a faster fix when subsequently powered on. The microcontroller geo-tags each set of sensor readings with GPS coordinates so that the values can be associated with a location.

The buoy also includes a DS3231 Real-time Clock (RTC) module that keeps track of time. The module includes a coin-cell battery that serves as a backup power source in case the main battery power gets disconnected. The microcontroller appends the time information to each set of sensor readings.

### Electronic components

GatorByte has other miscellaneous electronic components that assist the data generators in carrying out their function. These components are widely available and can be sourced from popular online stores. The high accessibility of these components has created an ecosystem of DIYers, stable firmware libraries, and technical support.

The GatorByte’s electronics components can be classified as – data generators (Section 2.3), microcontroller board, communications modules (Cellular module and Bluetooth module), power supply and management system (Li-Po battery and power control switches), and local data storage. The microcontroller executes the firmware procedures, collects the data from the data generators, saves the data on the local storage, and transmits the same data to the cloud-based server.

The electronic components in the GatorByte platform are described below.

#### Microcontroller board

The GatorByte buoy uses Arduino MKR NB1500, which is an LTE-M (Long-term Evolution-Machine Type Communication) and NB-IOT (Narrow-band Internet of Things) capable microcontroller board. Alternatively, for regions of the globe otherwise covered by GSM and 3G networks, the MKR GSM1400 microcontroller board can be used. GSM1400 and NB1500 use the same microcontroller (SAMD21 Cortex M0+), hence these can be used interchangeably (depending on the availability of cellular services) with only minor adjustments to the GatorByte firmware.

NB1500 supports multiple serial communication protocols (I^2^C, SPI, and UART) which makes it flexible and can be easily adapted for use with a variety of sensors and peripherals. The NB1500 shares processor architecture with numerous microcontrollers made by other manufacturers. Hence, it can be swapped with another Arduino-compatible microcontroller by making minor changes to the hardware and firmware [21].

#### Communications modules

The NB1500 has an integrated cellular module (u-blox SARA-R410M) that enables transmitting data to a remote server over the Internet. The cellular module supports LTE-M and NB-IoT technologies, which makes sending and receiving data more power-efficient, more resilient to errors, and less prone to delays. This directly translates to an extended operational life of the buoy. NB-IoT is available in most countries in North America, Europe, Australia, and Asia. While LTE-M is only available in North America, Europe, and Australia. Hence, GatorByte can be deployed on most continents.

The buoy also includes a Bluetooth module (HC-05 or AT-09). This module enables wireless interfacing of the buoy with a Bluetooth device in the field for data offloading and diagnostics.

The GatorByte uses an internal 4G antenna for cellular data transmission and a patch antenna for the GPS module. An inexpensive waterproof 2-in-1 antenna (4G and GPS) can be mounted externally on the dry (top) side of the housing to augment signal strength. The cellular signal strength declines sharply underwater; hence, the antenna needs to be above the water surface for optimal transmission.

#### Power supply, control, and management system

The Arduino NB1500 requires a power supply of 3.3 V to 5 V, hence a 3.7 V Lithium-Polymer (Li-Po) battery can be used to power the buoy.

A contactless power switching system has been implemented using two magnetic reed switches and a Dual Pole Dual Throw (2-coil latching, DPDT) relay. Bringing a magnet close to one of the reed switches turns on the GatorByte and bringing the magnet close to the other reed switch turns the GatorByte off. The locations of the reed switches are indicated on the lid by two side-by-side raised ovals.

A few power-saving features such as deep-sleep mode, Light Emitting Diode (LED) power control, cellular module power control, etc., have been implemented at the hardware and firmware levels (discussed in the following sections) which make the buoy well-suited for power-limited and long-term deployments [22], [23].

#### Data storage

The GatorByte platform uses a micro-SD breakout board to locally store water quality data. This module is 3 V–5 V tolerant, is compatible with 3.3 V logic microcontrollers, and uses Serial Peripheral Interface (SPI) protocol to communicate with the microcontroller. This module allows saving data as a backup in an event of a network outage or server unavailability. It is recommended to use a high-endurance, Class 10 microSD HC card with a storage capacity of 4 GB to 16 GB from a trusted brand as these are resilient and reliable. Inexpensive, lower-quality micro-SD cards have more frequent incidents of failure and data corruption.

In summary:

• The GatorByte’s enclosure is 3D printed using ASA filament and is post-processed with Acetone vapors. The enclosure uses O-rings and spar urethane spray coats for preventing water leakages.
• The enclosure is highly customizable and can be tailored according to individual monitoring requirements.
• The PBCs are modular, allowing users to easily alter and customize the buoy by adding or removing sensors or other electronic peripherals according to their sensing needs.

## Design and software files

The following section lists and describes the source files for the CAD designs (enclosure and PCB), and the software (firmware, cloud server, and dashboard).

### Enclosure CAD files

Table 2 lists the enclosure’s CAD files.

**Table 2:** Buoy’s CAD (SolidWorks Part file) and STL files.

Design file folder/name	Description
/hw/bottom.sldprt	Editable CAD file for enclosure bottom
/hw/sensor_thread_hole.sldprt	The threaded hole where the sensor screw-in on the buoy.
/hw/assembled_bottom.sldprt	Assembled bottom enclosure with sensor threaded holes mated with the bottom buoy.
/hw/lid.sldprt	Editable CAD file for enclosure lid
/hw/adapter_[SENSOR NAME].sldprt*	½ inch (1.27 cm) threaded adapters to attach pH, EC, DO, and temperature sensor probes to the buoy.
/hw/plug.sldprt	½ inch (1.27 cm) threaded plugs are to be used when no sensor is inserted in one of the four sensor holes.
/hw/stalk_fastner.sldprt	Fasteners to attach Stalk PCB to the underside of the enclosure lid.

*Replace [SENSOR_NAME] with ‘ph’, ‘rtd’, ‘ec’, or ‘dox’.

#Corresponding STL files are also included with the same file name.

#These files have been shared (https://doi.org/10.17632/gjbkxb74dw.1) under CERN OHL v2.0 license. The license is included in the repository.

### Firmware

Table 3a, Table 3b list the firmware and server software files.

**Table 3a:** Microcontroller firmware and firmware library.

Directory	Design file name/folder	File type	Description
Buoy firmware	/fw/NB1500/*	Firmware files	This folder contains all the files and folders of the Platform IO project. Platform IO is an extension for Microsoft VS Code that provides an Integrated Development Environment (IDE) for Arduino NB1500 firmware development and deployment.
GatorByte library	/library/GatorByte/*	GatorByte library	This folder contains the GatorByte library containing sub-libraries that enable modularity in the firmware design. The code.zip files already contain the GatorByte library in/lib/GatorByte folder.

#These files have been shared (https://doi.org/10.17632/y2gc2fwj5b.1) under the GNU GPL v3.0 license. The license is included in the repository.

**Table 3b:** Server code, dashboard code, and MQTT broker.

Directory	Design file name/folder	File type	Description
Dashboard web application	/sw/dashboard/*	JavaScript web application	This folder contains the source code for the GatorByte platform’s web dashboard developed using JS, HTML, and CSS.
Server application	/sw/server/*	NodeJS web application	This folder contains an Express NodeJS server application that serves data to the dashboard application and listens to sensor data updates. Issue command “npm -i” to install dependencies.
MQTT broker	/sw/broker/*	JavaScript MQTT broker	This folder contains a JS MQTT broker application, built using Aedes JS. A broker acts as the handler of MQTT topic publications and subscriptions. Issue command “npm -i” to install dependencies.

#These files have been shared (https://doi.org/10.17632/csf6hhvy3b.1) under GNU AGPL v3.0 license. The license is included in the repository.

### PCB files

Table 4 shows the list of CAD files related to the PCBs.

**Table 4:** GatorByte PCB schematic and board layout files.

Design file name/folder	File type	Description
/pcb/stalk.sch	EAGLE schematic	Stalk PCB schematic
/pcb/stalk.brd	EAGLE board layout	Stalk PCB board layout
/pcb/uc_hat.sch	EAGLE schematic	Microcontroller hat schematic
/pcb/uc_hat.brd	EAGLE board layout	Microcontroller hat board layout
/pcb/baseboard.sch	EAGEAGLE schematic	Sensor baseboard schematic
/pcb/baseboard.brd	EAGLE board layout	Sensor baseboard schematic

#These files have been shared (https://doi.org/10.17632/gjbkxb74dw.1) under CERN OHL v2.0 license. The license is included in the repository.

## Bill of materials (BOM)

The following tables (Table 5, Table 6, Table 7) list all the components needed to build a GatorByte buoy. Labor costs are included in Table 8.

**Table 5:** BOM for GatorByte enclosure.

Part	Quantity	Cost per unit (USD)	Total cost (USD)	Supplier
ASA filament 1.75 mm	1	$10	$30	Amazon
Spar Urethane spray canister	1	$2.00	$14	Amazon
Flex seal canister	1	$2.00	$13	Amazon
Press-fit barrel screws	4	$0.45	$45	McMaster-Carr
M2 × 7 mm screws	2	$0.10	$10	Amazon
Buoy-sensor interface O-ring	4	$0.06	$6.33	McMaster-Carr
Buoy enclosure interface O-ring	4	$1.05	$10.53	McMaster-Carr
Epoxy glue	1	$1.00	$19.99	Amazon
Acetone	1	$1.00	$9.99	Amazon

**Table 6:** BOM for GatorByte electronic components.

Part	Quantity	Cost per unit (USD)	Total cost (USD)	Supplier
Arduino NB1500	1	$85	$85	Arduino store
NB-IoT antenna	1	$1.8	$9	Amazon
AT&T Global IoT SIM	1	$3	$3 kit + ($2/month)	AT&T marketplace
AHT-10 sensor	1	$3.2	$16	Amazon
DS3231 RTC	1	$1	$10	Amazon
3.3 V to 5 V booster	1	$1	$10	AliExpress
AT24C256 EEPROM	1	$3.5	$7	AliExpress
SN74HC595 shift register	2	$0.36	0.72	AliExpress
n-channel SOT-23 MOSFET	30	$0.02	$0.6	LCSC
0805 10 k ohm resistor	2	$0.01	$0.02	LCSC
0805 33 k ohm resistor	20	$0.01	$0.2	LCSC
0805 1 K ohm resistor	3	$0.01	$0.03	LCSC
2-Pin JST connector	1	$0.01	$2	AliExpress
Adafruit neo-6 m GPS module	1	$20	$20	Amazon
SparkFun level-shifting SD module	1	$6	$6	SparkFun
16 GB micro-SD card	1	$3.30	$16.50	Amazon
AT-09 Bluetooth module	1	$3.75	$15	Amazon
MPU6050 accelerometer	1	$2.10	$21	AliExpress
pH OEM IC	1	$41.40	$41.40	Atlas Scientific
pH mini probe	1	$56.40	$56.40	Atlas Scientific
EC OEM IC	1	$58.50	$58.50	Atlas Scientific
EC K 1.0 mini probe	1	$114.70	$114.70	Atlas Scientific
RTD OEM IC	1	$31.70	$31.70	Atlas Scientific
RTD PT-1000 probe	1	$22.20	$22.20	Atlas Scientific
DO OEM IC	1	$47.70	$47.70	Atlas Scientific
DO mini probe	1	$124.85	$124.85	Atlas Scientific
TX2-LT-3V-TH Relay	1	$4.79	$4.79	Mouser
Reed switches	2	$5.98	$12	Mouser
RGB 5050	1	$0.04	$0.04	LCSC
TP4056 Li-Po battery charging module	1	$1	$8	Amazon
Li-Po battery (4500 mAh)	1	$17	$17	Amazon
Silicone conformal coating canister	1	$22.42	$22.42	Amazon

**Table 7:** BOM for connectors in GatorByte.

Part	Quantity	Cost per unit (USD)	Total cost (USD)	Supplier
JST connector shroud	1	$0.01	$0.5	AliExpress
IDC cable	1	$4.79	$4.79	Amazon
IDC shroud	1	$4.79	$4.79	Amazon
IDC connector	1	$4.79	$4.79	Amazon
Jumper	1	$0.02	$0.02	Amazon
20-Pin female headers	4	$10.99	$10.99	Amazon
40-Pin male headers	4	$10.99	$10.99	Amazon
10-Pin right-angled male headers	1	$6.99	$6.99	Amazon/AliExpress
2-Pin screw terminals	1	$9.99	$9.99	Amazon

**Table 8:** Labor costs.

Part	Hours	Cost (USD) ($15/hr rate)	Skillset requirement
Soldering PCB	3	$45	Knowledge of electronics and soldering Surface Mount Devices (SMD)
Enclosure 3D printing, cleaning, and post-processing	2	$30	Using CAD and slicing software
Programming microcontroller	1	$15	Programming experience
Assembly of the GatorByte	1	$15	N/A

## Build instructions

The description of steps for producing a GatorByte buoy is below. The section delineates steps to customize and fabricate the PCBs, enclosure, and firmware.

### Customizing PCB layout and fabrication

GatorByte’s electronics` design is modular and constitutes three PCBs – the microcontroller hat, sensor baseboard, and the stalk:

a. To sustain compatibility between the modular PCBs, some dimensions are standardized. Moreover, the universal GatorByte interface (see Fig. 4 and Fig. 5) should not be altered.
b. To add/remove sensors or peripherals, edit the PCB design files (mentioned in section 3.3) using Autodesk EAGLE™.
c. Finalized PCB designs can be exported as Gerber files (packaged as a.zip file), and uploaded to online PCB manufacturers, like PCBway.com or JLCPCB.com, that will produce and ship worldwide.
d. Once the PCBs are manufactured, solder the components onto the three PCBs. This step requires some experience with soldering and additional tools, for example, solder, solder gun, heat gun, desoldering station, wire clippers, and tweezers.
e. Soldering Surface-mount Devices (SMD) like SOT23 MOSFETs, 0805 resistors, and SN75HC595D ICs require additional expertise. The task of soldering SMDs can be off-loaded to PCB manufacturing companies mentioned above for an additional fee that covers soldering and parts.
f. The assembled PCBs must be cleaned to remove any residual flux on the board since this can cause a high-impedance short circuit and cause sensitive sensors like pH probes to report incorrect values. The flux cleaning can be done in the following ways:
  a. Clean the PCB by soaking it in Isopropyl alcohol (IPA) for ∼ 30 to 45 min, stirring occasionally. A brush can be used to remove stubborn flux residue. Finally, rinse the PCB with IPA and dry it with a hot air gun or allow it to air dry.
  b. As an alternative, an ultrasonic cleaner can be used with a PCB cleaning solution which takes ∼ 5 to 8 min per PCB and is more reliable compared to dipping PCBs in IPA.
g. Once the PCBs are fully dry. Apply a coat of silicone conformal coating and let the coat cure for 24 h. The conformal coating protects sensitive electronic components from moisture inside the buoy. It is recommended to apply a second coat once the first coat has fully cured.

### Customizing enclosure and fabrication

GatorByte buoy’s enclosure has six parts (see Section 3.1) that are customizable (if necessary) and may be printed using a hobby-grade 3D printer. The parts are:

a. Bottom
b. Lid
c. Sensor adapters
d. Stalk fastener
e. Screw hole caps
f. Plug (if required)

The steps for customizing and fabricating the enclosure are listed below:

a. If the user needs to add/remove a sensor or alter the dimensions of the enclosure, the enclosure design may need revision. Edit the files (mentioned in Section 3.1) and export the files in STL format. The files *bottom.sldprt* and *sensor_thread_hole.sldprt* need to be assembled before being exported to STL file format).
b. If no customization is required, the provided STL files (mentioned in section 3.1 may be used as is with slicer software to create GCODE files.
c. Once the parts have been printed, it is recommended to perform acetone treatment on the enclosure parts as described in Section 2.1.

### Customizing firmware and flashing to the microcontroller

The process for updating and flashing the firmware to the microcontroller is listed below.

a. Install Microsoft VS Code and then install the platformIO VS Code extension [24]. Configure platformIO and install the toolchain for Arduino NB1500.
b. If needed, users can make changes to the procedures in the firmware.
c. The GatorByte Arduino library provides a user-friendly interface to interact with the sensors and other components of the buoy. The customization of the firmware will involve changing the functions provided by the GatorByte library and changing the sequence of when those functions are called.
d. With the NB1500 connected to the computer, flash the firmware to the microcontroller using platformIO’s interface.
e. The platformIO serial monitor (SM) provides live logs of the microcontroller. The firmware logs every action that it takes, hence the SM can help with troubleshooting any issues, or can just be used to monitor the operation of the firmware.

### Assembling all components

**Fig. 7:**
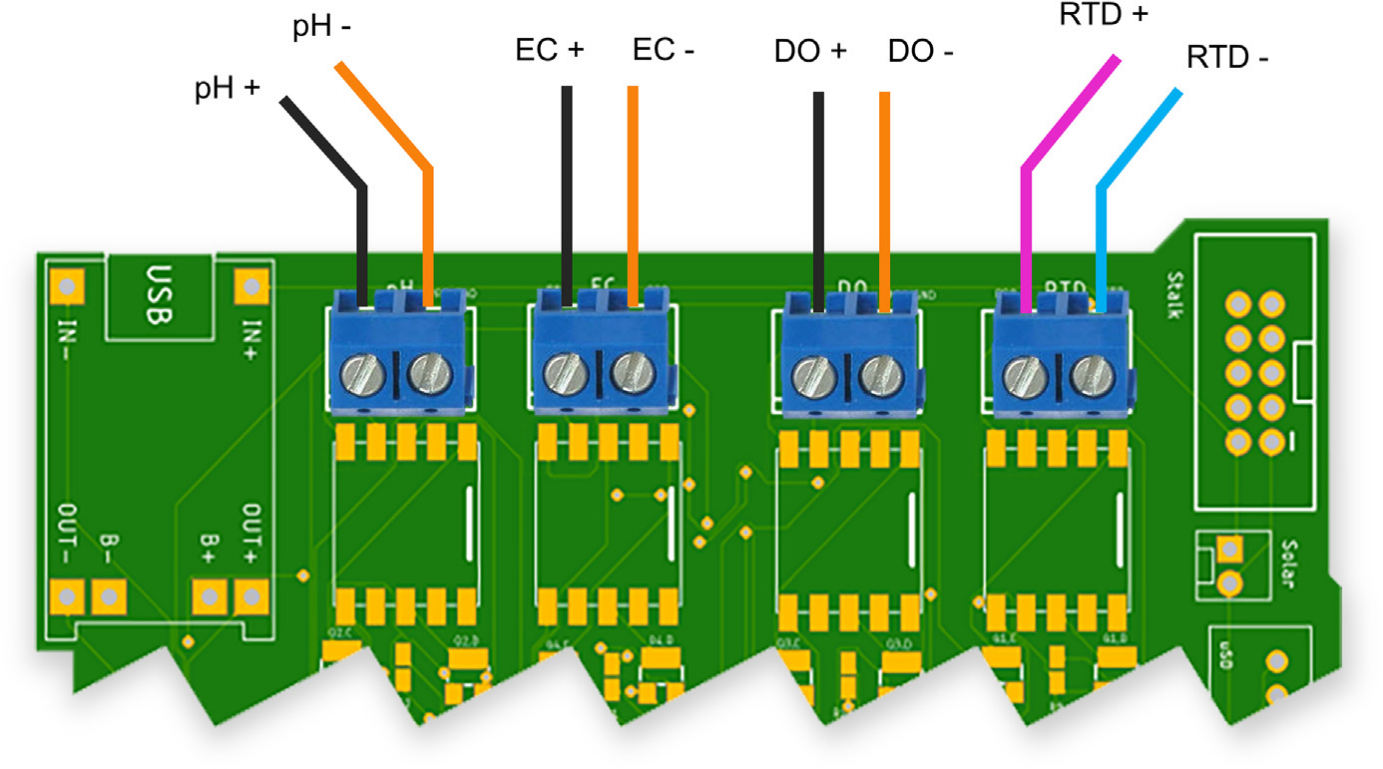
**Sensor interface wiring schematic –** The illustration above shows the sensor wire connection schematic. The wires are color-coded per their physical appearance.
e. The illustration in Fig. 7 shows two-wire screw terminals and the connection schema for the sensor probes.
f. Using stalk fasteners, attach the stalk PCB to the inner surface of the lid enclosure using M2 7 mm screws.
g. Using an IDC cable, connect the stalk PCB to the sensor baseboard PCB.
h. Attach the microcontroller hat to the sensor baseboard PCB at the universal GatorByte interface.
i. Connect the battery’s JST connector to the sensor baseboard and connect the JST connector from the sensor baseboard to the microcontroller hat.
j. Place the plastic jumper piece on the off position on the microcontroller hat until the buoy is ready to be used.
k. Attach the NB-IoT antenna to the NB1500 microcontroller, and the GPS antenna to the GPS module.
l. Place the assembled PCBs in the slot within the buoy enclosure.
m. Perform a buoyancy test in a bucket of water.
  i. Add weights to the ballasts in the buoy enclosure to correct the orientation of the buoy.
  ii. Once the buoyancy test is complete, close the lid using the press-fit screws.
n. A multiday experiment to test the buoy’s ability to keep water out is recommended. Leave the buoy in a bucket of water for at least 48 h. If there is a leak, ensure the O-rings are properly installed, reapply the Teflon tape, and that the sensor adapters are screwed in place tightly. Also ensure, at least 5 coats of urethane spray have been applied on at least the inner surface of the buoy enclosure’s bottom and lid enclosure. Repeat this experiment until the leaking stops.

The steps for assembling the components are listed below:

a. To prepare the sensors for installation:
  i. Using epoxy glue, adhere sensor probes to the sensor adapters. This interface must be waterproof.
  ii. Apply Teflon tape to the threads of the sensor adapters.
  iii. Cut the sensor probe wires to the appropriate length (∼10 – 15 cm)
  iv. Strip and tin the exposed wires.
b. Insert the sensor-buoy interface O-rings and the enclosure interface O-ring. The enclosure interface O-ring may require adhesive for proper seating.
c. Screw in sensors (already in sensor adapters) into the buoy. Ensure that the adapters are screwed in tightly to prevent any water leaks.
d. Connect the sensor wires to the respective screw terminals (Fig. 7).

## Operation instructions

This section describes typical maintenance tips for the buoy, including the steps to calibrate sensors, pre-deployment configuration, and field deployment of the buoy.

### General maintenance

We recommend users exercise typical precautions and conduct normal maintenance practices that they would with any other environmental monitoring system or sensor.

Buoy maintenance includes many aspects, including sensor maintenance (including calibration), an inspection of the enclosure, O-rings replacements, and charging the battery. These are discussed in the following sections.

The following list lays out general considerations for proper maintenance of the device.

1. Users are recommended to check sensor calibrations, inspect the enclosure for cracks and degradation, and charge the battery before every survey.
2. The sensors (the most expensive part of the system) have a shelf life of ∼5 years, but when deployed often, will need replacement every 1 to 2 years.
3. Li-Po batteries degrade over time, but their life depends on the number of discharge cycles. We recommend replacing batteries at least every year to ensure the battery can provide sufficient power for the intended duration of the survey.
4. The O-rings used in the GatorByte are made from Buna-N and are resistant to mild chemicals and water. We expect the O-rings to outlast the sensors, the enclosure, and the electronics and will not need replacement unless they have been damaged due to other factors.

### Calibration of sensors

Environmental sensors must be calibrated before deployment. Typically, sensors lose calibration and drift over time, and hence, periodic calibration of the sensors should be performed before each deployment. GatorByte uses environmental sensors from Atlas Scientific which have varied calibration requirements. The requirements are included in Table 9.

**Table 9:** Atlas Scientific sensor’s calibration requirements.

Sensor	Requirement
Mini Lab Grade **pH** probe	Every year for the first 2 years; Every 3 months after that
Mini Lab Grade **DO** probe	Every 6 months
Mini Lab Grade **EC** K1.0 probe	Every 10 years
PT-1000 **RTD** probe	Not required

**Fig. 8:**
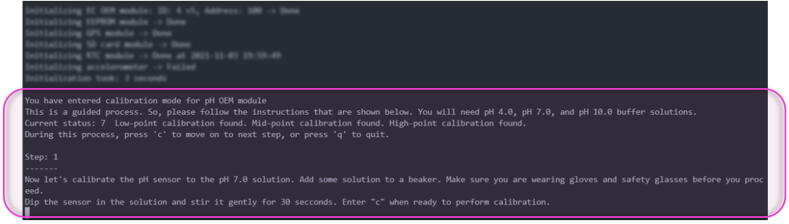
**Guided calibration process –** The image above shows the intuitive and easy-to-follow calibration instructions baked into the GatorByte’s firmware library.

Atlas Scientific sensor probes interface with the GatorByte via OEM interface Integrated Circuits (ICs), also sourced from Atlas Scientific. These OEM circuits convert weak electrical signals (voltages, currents, changes in resistances, etc.) into numerical values representing either pH, electroconductivity, dissolved oxygen, or temperature. The OEM circuits also provide a user-friendly interface to perform calibration of the sensors. The users need not fiddle with equations to carry out the calibration. Instead, the OEM circuits perform the calculations in the background. Calibration involves sending a 4-byte number corresponding to the calibration solution being used. The four bytes need to be written to specific registers on the OEM circuits. The GatorByte firmware provides an abstraction so that users need not concern themselves with knowing which registers to write to. The firmware provides a guided calibration procedure (Fig. 8) for the sensors that require calibration (pH, D.O., and E.C. sensors), and users can perform calibration with a few keystrokes.

The RTD temperature sensor in GatorByte uses the correlation between the resistance of Platinum metal and the temperature. This relationship is constant and does not drift with time, and hence the RTD sensor does not need calibration. However, it is good practice to periodically validate the temperature probe readings.

To perform calibration, it is recommended to unscrew the sensors from the GatorByte’s enclosure for ease of handling and safety.

a. Connect the sensors to a completely assembled GatorByte circuitry (Microcontroller hat, stalk, and sensor baseboard). Connect the microcontroller to a computer using a USB cable.
b. Open a “Serial monitor” (SM) software on the computer, select the port the microcontroller is connected to, and ensure you can see the microcontroller’s diagnostic information displayed on the SM.
c. In calibration mode, the normal operation of the device will cease. Step-by-step instructions will be displayed on SM to assist with the calibration procedure.
d. Follow the prompts on SM and repeat for the other sensors.
e. User commands while in calibration mode are included in Table 10.

**Table 10:** Calibration mode commands.

Command	Description
**c**	**Continue** to the next step
**s**	**Skip** the current step
**r**	**Read** the sensor value
**w**	**Read** 30 consecutive values
**x**	**Delete** the existing calibration
**/**	**Show** current calibration status
**q**	**Quit** the calibration mode
**+**	**Power on**
**a**	**Activate** sensing subsystem
**–**	**Power off**
**d**	**Deactivate** sensing subsystem

### Device configuration and operation modes

The GatorByte platform allows modifying a buoy’s configuration in three ways.

a. Edit config.ini file on the SD card (pre-deployment)
b. Update using a Bluetooth-enabled smartphone (during deployment)
c. Update using the web dashboard (post-deployment)

Any updates made using the web dashboard or a Bluetooth device will edit the config.ini file on the micro-SD card present on the GatorByte buoy. Editing the config.ini file allows the user to adjust the sleep duration and sampling frequency (resolution), enable or disable sensor/peripherals, and change operation modes.

All available operation modes on the GatorByte platform are listed in Table 11 below.

**Table 11:** Operation modes of GatorByte buoy.

Mode	Description
**real-time**	**Real-time** reporting, SD logging
**offline**	**No** reporting, SD logging
**command**	**Paused** operation, listens for user commands via Serial Monitor
**dummy**	**Dummy readings;** useful for diagnostics

### Powering the device on/off.

GatorByte can be powered on or off without opening the watertight enclosure by following the steps below.

a. To turn on the device, bring the magnet close to the marking “**|**” on the top side of the lid. To turn the device off, bring the magnet close to the ‘O’ marking.
b. If the device has been non-operational for an extended period, the GPS module will need a couple of minutes to acquire location the first time. Place the device outdoors until the green LED on the GPS module starts blinking every 2 s.
c. Ensure all wire connections are made and the screws are in place; release the GatorByte for the survey.
d. Monitor the latest position and water quality data on the dashboard. If the battery level drops below 15%, collect the device before the battery runs out to ensure that the device can be located and recovered.

### Deployment instructions

Ensure the configuration file on the micro-SD card (Section 6.2) is updated before deploying. At the deployment site, use the pre-survey checklists as laid out in Table 12 and Table 13 to ensure that the buoy is configured correctly and that the peripherals and sensors are working as expected.

**Table 12:** Pre-survey hardware checks.

Action	Description
Charge battery	Charge two batteries the day before conducting a survey. Use the second battery as a spare.
Unload SD card	Back up any old data on the SD card on a computer.
Update configuration file	Create/update the configuration file on the SD card with information about the survey name, survey date, sampling frequency, sleep duration, et cetera.
Synchronize RTC	Ensure the RTC clock reports accurate time. If the clock is out of sync. rtc.sync() function syncs the RTC’s time with the time of flashing the firmware.
Install SIM card and antenna	Ensure the SIM is properly seated. Bring a spare antenna.
Install GPS antenna	Install a small-form-factor internal GPS patch antenna. Bring a spare to the site.
Apply conformal coating	If not already done, air dry the PCBs and then apply conformal coating on them.
Set power jumper to “On”	Ensure that the jumper on the microcontroller hat is set to the “On” position.
PCB connections	Connect microcontroller hat, sensor baseboard, and stalk PCBs.

**Table 13:** Pre-survey software checks.

Action	Description
Update firmware	If needed, update the firmware, and flash the new firmware to the microcontroller.
Run diagnostics code	Add gb.diagnostics() to the setup block of the code and ensure all peripherals are reporting.
Dry run	Perform a test run of the firmware to ensure the code is working as expected

## Validation and characterization

This section describes the validation and characterization of the GatorByte buoy. This section includes the following subsections:

a. Buoy operation
b. Power budgeting
c. Sensor data validation

The buoy was deployed in two scenarios to assess its performance.

a. Multiday survey at a pond at Center of Aquatic and Invasive Plants (CAIP), Gainesville FL
b. 2-hour survey at Sweetwater branch creek (SWB), Gainesville FL

### Buoy operation

When operating in “real-time” mode, GatorByte buoy reports the data to the backend server hosted on Amazon Web Services (AWS) in real-time as well as saves the data locally on the micro-SD card. GatorByte uses Comma-separated Values (CSV) format to transmit and store the data.

The buoy reports the data as soon as it takes sensor reading using MQTT (MQ Telemetry Transport) instead of HTTP (Hyper Text Transfer Protocol). MQTT is more suitable for IoT applications due to smaller network overhead and quicker data transfers [25]. Using MQTT, the buoy takes ∼ 10 ms to transfer the sensor data while HTTP takes ∼ 50 ms to transfer the same data.

If the buoy enters an area with no cellular service or low cellular signal, besides logging the data to the micro-SD card, it also queues the data to the micro-SD card for the data upload to be reattempted as soon as the buoy regains cellular connectivity in the future.

The GatorByte can alternatively be operated in “offline” mode. In this mode, the cellular MODEM stays off and the buoy does not attempt to upload the sensor data to the server. The data will have to be manually uploaded to the GatorByte dashboard using the “upload” feature on the dashboard.

The sensor data is visualized on the GatorByte dashboard in real-time using a map and charts unless the buoy is operating in “offline mode”. The dashboard can also be used to change the buoy’s configuration parameters, for example, sampling frequency, operation mode, sleep duration, etc.

### Power budgeting

Average power consumption of the GatorByte buoy while in awake, idle, and asleep modes is included in Table 14. A 4500 mAh battery was used to conduct the surveys at CAIP and SWB. The battery lasted ∼45 h at a 5-minute sampling frequency. The average current consumption of each electronic peripheral on GatorByte is shown in Table 15.

**Table 14:** Avg. current and power consumption of the GatorByte buoy as a system.

Scenario	Avg. Current (mA)	Avg. Power at 4 V (mW)	
Awake	40	160	
Asleep	1.5	6	
Idle mode	15	60

**Table 15:** Avg. current and power consumption of individual electronic components in the buoy.

Device	Avg. Current (mA)	Avg. Power at 4 V (mW)
NB1500 (MODEM on)	75	300
NB1500 (in sleep mode)	0.5	2
AT-09 Bluetooth module	40	160
MP6050 Accelerometer	5	20
Neo-6m GPS module	65	260
AT24C EEPROM	3	12
DS3231 RTC	0.5	2
micro-SD module	30	120
RGB (set to white)	10	40
pH OEM IC	3	12
EC OEM IC (up to 2000 µS/cm)	15	60
DO OEM IC	2.5	10
RTD OEM IC	5	20

### Sensor data validation

The GatorByte buoy was deployed at two trial water quality surveys in Gainesville, Florida. Both locations are urban freshwater water bodies. The following subsections describe the survey trials and their outcomes.

Pre- and post-survey calibrations were performed to ensure the accuracy of the data. However, a compensable error was discovered in the Sweetwater Branch’s pH sensor values during post-survey calibration. It was discovered that stray voltages on the EC sensor and DO sensor interfere with the pH sensor through the water. As a result, the EC sensor pulled the pH values a few fractions of pH units lower than the true value, while the DO sensor pulled the pH values higher than the true value. Their combined effect resulted in pH values being 0.2 to 0.3 units higher than the true value. The true value was obtained using two reference handheld sensor systems – Hanna HI9813 ($230, single-point calibration) [26], and OTT Hydrolab HL4 (∼$15,000, multi-point calibration) [27].

#### Center for aquatic and invasive plants (CAIP) survey

##### Location

The GatorByte was deployed at CAIP to demonstrate its capabilities in July 2022. CAIP has 9 ponds teeming with aquatic life including vegetation, fish, turtles, snails, and frogs. The pond that was used for the trial has a surface area of 2,000 sq. meters.

CAIP is located on the outskirts of Gainesville city, and hence, cellular coverage is spotty in the vicinity. GatorByte was initially configured to run in “real-time” mode, however, the buoy switched to “offline” mode to conserve battery and prevent the microcontroller from freezing the operation. Hence, recorded data was manually extracted from the onboard micro-SD card.

The buoy was tethered with a 1-meter-long fishing line for quickly and easily collecting the buoy at the end of the survey.

##### Objective of the deployment

The deployment’s main goal was to evaluate the battery life and operational duration and compare the observations with the estimated battery life. Moreover, the location does not have reliable network availability, hence GatorByte’s fault tolerance against network-related issues, including software hangs, was tested. The location witnessed moderate rain throughout the survey and as a result, the enclosure’s water-tight seals were tested as well.

##### Data and visualization

**Fig. 9:**
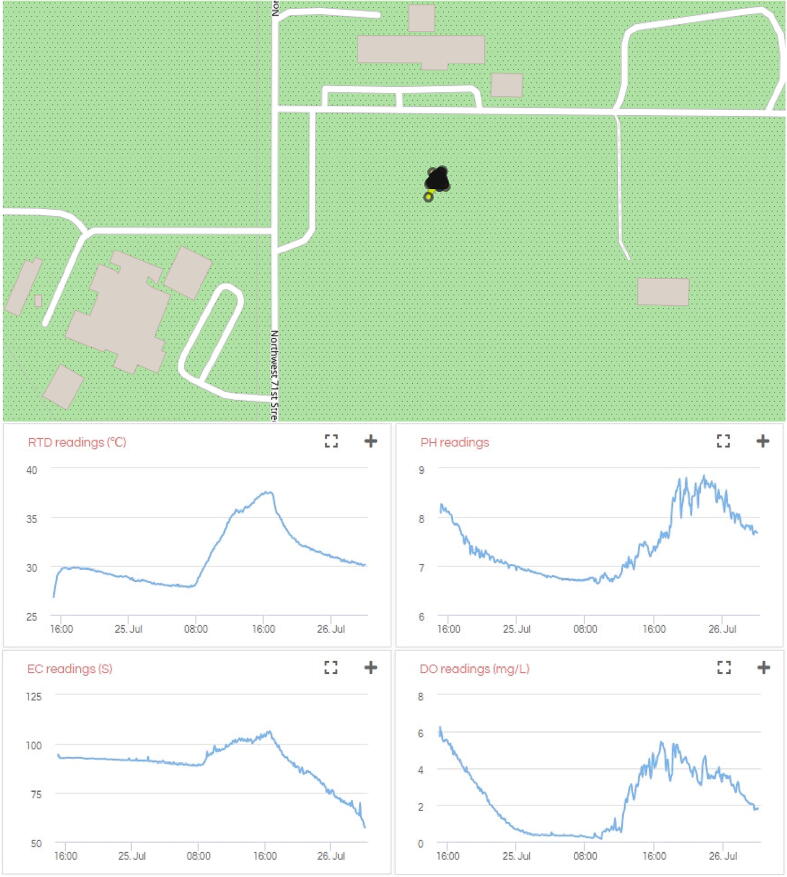
**CAIP survey data visualization –**Map and charts showing the data from the water quality survey at CAIP, Gainesville, FL, USA.

The data collected from GPS, pH, DO, EC, and RTD sensors can be downloaded in JSON format using the URL https://api.ezbean-lab.com/v3/gatorbyte/data/get?device_id=caip&survey_id=hwx. The data is visualized in Fig. 9.

##### Analysis of data and survey results

In addition to rain, the location was being pumped with freshwater by the CAIP to restore the water level. All the sensors show diurnal cycles in the data visualization and show variations as expected. The high-frequency components (short-period jumps and dips in pH and DO data) were attributed to the changes in the cloud cover. When the sun was blocked by the clouds, the DO went down, and vice-versa.

##### Conclusion

The survey lasted 37 h at a 5-minute sampling frequency. The sensor values show expected variations in response to diurnal cycles, weather variations, cloud cover, et cetera. The pH and DO values show some noise, however, a filtering technique can be used to smoothen the curve. The map does not show any significant movement since the buoy was tethered to the edge of the pond.

#### Sweetwater Branch Creek, Gainesville (SWB)

##### Location

This survey was conducted at Sweetwater Branch (SWB) Creek in Gainesville, FL. SWB is a small creek that ends up merging with Sweetwater Reserve. SWB is downstream of Gainesville’s water treatment plant. The weather was warm and sunny with low cloud cover. However, SWB is mostly shaded by the trees around the creek.

The creek’s depth varied between a few centimeters and a meter on the day of the survey. The creek’s bed is lined with sand with some vegetation and broken tree branches. Hydrolab HL4 was used as a reference sensor for this survey. The sensor values are plotted in the charts below.

##### Objective of the deployment

The primary objective of this deployment was to evaluate the sensor readings, the accuracy of the GPS module, network signal strength and ability of the communications subsystem to recover from network-related errors, and the ability of the enclosure to keep water from ingress into the buoy in an urban stream setting.

OTT Hydrolab HL4 sonde was used in conjunction with the GatorByte as a reference sensor. The sonde recorded pH, temperature (RTD), and electroconductivity water quality metrics.

##### Data and visualization

**Fig. 10:**
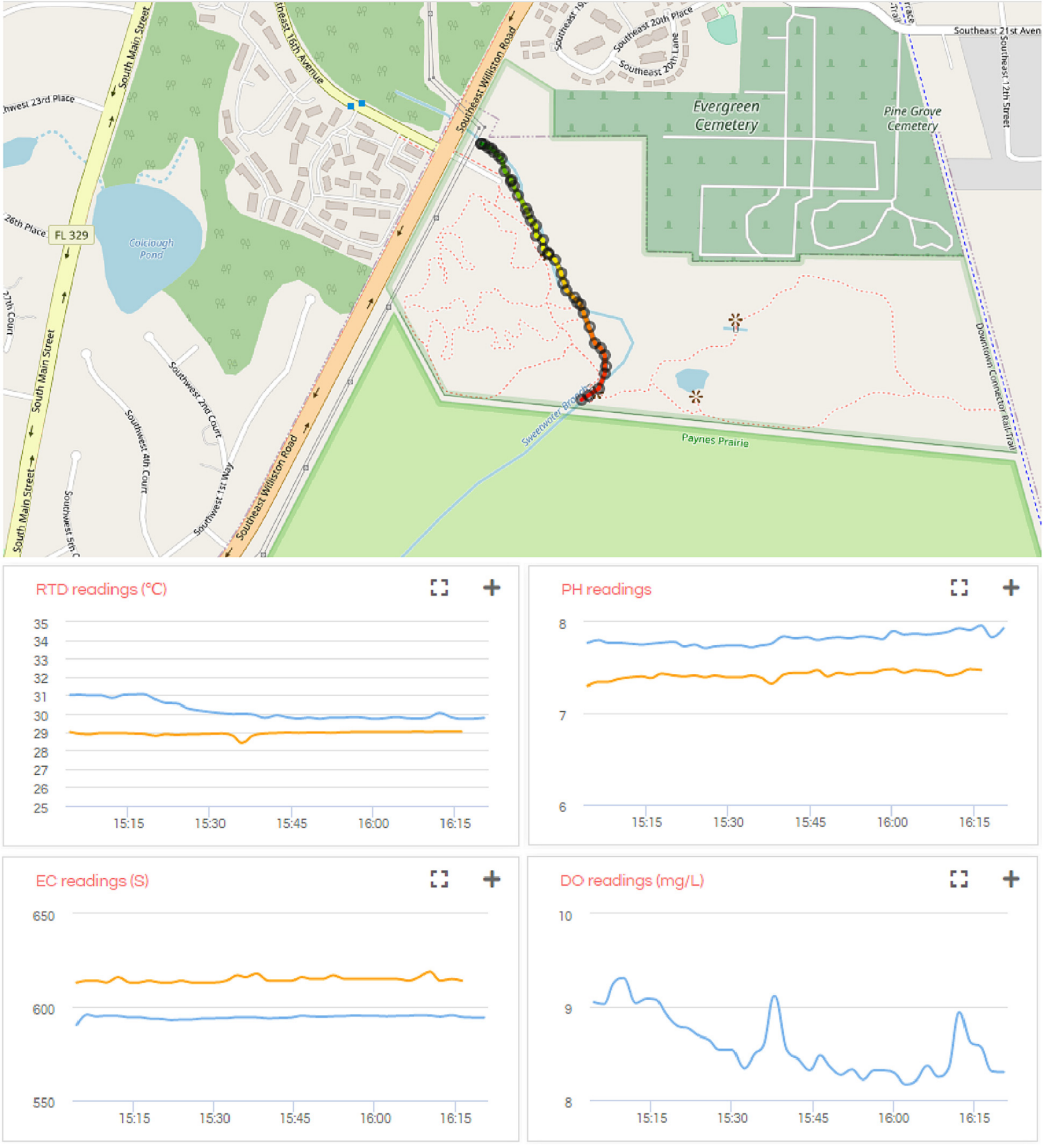
**SWB survey data visualization –**Map and charts showing the data from the water quality survey at SWB, Gainesville, FL, USA. The blue lines show the data reported by GatorByte, while orange show the data collected by Hydrolab HL4 sonde. (For interpretation of the references to color in this figure legend, the reader is referred to the web version of this article.)

The data collected from GPS, pH, DO, EC, and RTD sensors on the GatorByte can be downloaded in JSON format using the URL https://api.ezbean-lab.com/v3/gatorbyte/data/get?device_id=gb-swb-val&survey_id=hwx. The GatorByte data is visualized in Fig. 10 in blue color.

The pH, RTD, and EC readings recorded by the Hydrolab HL4 can be downloaded in JSON format by visiting the following URL https://api.ezbean-lab.com/v3/gatorbyte/data/get?device_id=gb-swb-val&survey_id=hwx&data_type=reference. This data is visualized in Fig. 10 in orange color.

##### Analysis of data and survey results

The GPS data visualized in Fig. 10 shows a close overlap with the stream’s path. The discrepancy in the southwest region of the stream is a result of inaccurate map tiles from OpenStreetMap. Map tiles from Google Maps align with the GPS data collected by the GatorByte buoy.

The data collected using the water quality sensors in the buoy show slight differences in temperature and electroconductivity readings, whereas the pH readings show a significant difference. Temperature readings from the buoy show a declining trend because the buoy was transported in an open box, while the HL4 sonde was transported to the location in a wet container. Hence, the sensor on the buoy took approximately 15 min to equilibrate with the stream water’s temperature. The discrepancy in the temperature readings was highest at 2 °C at the start of the survey and stabilized at a 1 °C difference. The difference in the electroconductivity readings was consistently around 10 µS/cm. The significant discrepancy in the pH readings was caused due to an electrical interference between the pH probe and electroconductivity and dissolved oxygen probes. The electroconductivity and dissolved oxygen probes did not turn off while the pH sensor was being read due to a hardware issue, which was resolved after the survey by employing MOSFETs to completely isolate the probes and prevent inter-sensor interference. The isolation was tested post-survey and was found to remove the discrepancies between the pH readings of the buoy and the HL4 sonde.

##### Conclusions

The survey lasted around 90 min and the buoy covered over 750 m. The visualized data showed no major changes in the water quality parameters during the survey. However, the values reported by buoy sensors were a little off compared to those of the OTT Hydrolab HL4. This discrepancy was identified as caused due to sensor drift. Periodic calibration will resolve the discrepancies in the sensor values.

## Declaration of Competing Interest

The authors declare that they have no known competing financial interests or personal relationships that could have appeared to influence the work reported in this paper.
